# A novel five-lncRNA signature panel improves high-risk survival prediction in patients with cholangiocarcinoma

**DOI:** 10.18632/aging.202446

**Published:** 2021-01-20

**Authors:** Xiaozai Xie, Yi Wang, Sina Zhang, Jialiang Li, Zhengping Yu, Xiwei Ding, Longyun Ye, Peirong Gong, Qiandong Zhu, Junjian Li, Ziyan Chen, Xinfei Yao, Zhiyong Du, Qiqiang Zeng, Hanbin Chen, Zhen Yang, Gang Chen

**Affiliations:** 1 Department of Hepatobiliary Surgery, The First Affiliated Hospital of Wenzhou Medical University, Wenzhou, China; 2 Key Laboratory of Diagnosis and Treatment of Severe Hepato-Pancreatic Diseases of Zhejiang Province, The First Affiliated Hospital of Wenzhou Medical University, Wenzhou, China; 3 Department of Epidemiology and Biostatistics, School of Public Health and Management, Wenzhou Medical University, Wenzhou, China; 4 School of Public Health, Inner Mongolia Medical University, Hohhot, China; 5 Department of Gastroenterology, The Affiliated Drum Tower Hospital of Nanjing University Medical School, Nanjing, China; 6 Department of Hepatobiliary Surgery, The Affiliated Cixi Hospital of Wenzhou Medical University, Ningbo, China; 7 Division of Clinical Medicine, First School of Clinical Medicine, Wenzhou Medical University, Wenzhou, China; 8 Department of Hepatobiliary Surgery, Shenzhen People’s Hospital, Shenzhen, China; 9 Department of Radiotherapy and Chemotherapy, The First Affiliated Hospital of Wenzhou Medical University, Wenzhou, China; 10 Department of Infectious Diseases, Shandong Provincial Hospital, Jinan, China

**Keywords:** cholangiocarcinoma, lncRNA, overall survival, prognosis prediction, risk-stratification

## Abstract

Cholangiocarcinoma (CCA) is a fatal disease with dismal survival rates. Long non-coding RNA (lncRNA) expression profiling as potential prognostic biomarkers play critical roles in tumor initiation, development, and poor prognosis. Identifying specific lncRNA to predict the prognosis of CCA patients in the early stages is very important for improving a patient’s survival. In the current study, we aimed to establish a novel risk-stratification lncRNA signature panel in CCA. The initial lncRNA discovery was identified in The Cancer Genome Atlas database (TCGA cohort). The Cox regression analysis was used to establish the lncRNA prognostic model and the receiver operating characteristic (ROC) curve analysis was performed to assess the specificity and sensitivity of the model. This was followed by independent validation of the lncRNA signature in the CCA patients from the First Affiliated Hospital of Wenzhou Medical University (WMU cohort). Furthermore, by using the Gene Ontology function and Kyoto Encyclopedia Gene and Genome pathway enrichment analysis, we explored the potential function of prognosis lncRNA. Finally, five lncRNA (HULC; AL359715.5; AC006504.8; AC090114.2; AP00943.4) were screened to establish the predictive model that significantly associated with poor overall survival(HR:4.879;95%CI,1.587-14.996;*p*=0.006). This five-lncRNA signature model showed excellent accuracy in the TCGA cohort (AUC=0.938), and also robustly predicted survival in the validation WMU cohort(AUC=0.816). Functional enrichment analysis suggested prognostic lncRNA was primarily associated with CCA-related biological processes. Our data established a novel lncRNA signature model for CCA risk-stratification and robust identification of CCA patients with poor molecular genotypes. Moreover, it revealed new molecular mechanisms of CCA.

## INTRODUCTION

Cholangiocarcinoma (CCA) is the second most common primary liver cancer after hepatocellular carcinoma, with incidence and mortality rates rising across the world [1, 2]. Despite surgery and liver transplantation being options for patients, the high recurrence rate leads to CCA patients’ median survival time of less than one year [3]. Moreover, whether adjuvant therapy after surgical resection is effective, because data about its overall efficacy and survival benefits are limited [4]. Clinicopathological factors of CCA such as grade and stage are strongly associated with prognosis and are also key factors determining the therapeutic regimen. However, even with similar clinical characteristics, the prognosis of CCA patients is significantly different. Therefore, it is important to identify efficient tumor features to help clinicians stratify high-risk patients and tailor personalized treatment regimens for improving treatment outcomes.

With the development of gene sequencing technology, there has been a growing interest in using gene expression signature for risk-stratification of cancer patients. Besides, anti-cancer drugs based on genetic research are developing rapidly [5–7]. Therefore, conducting further studies on CCA-related genes and epigenetic markers such as long noncoding RNA (lncRNA) to guide personalized treatment in order to reduce recurrence and improve survival rate is warranted [8]. In the past decades, lots of evidence has suggested that lncRNA is strongly related to tumor occurrence, metastasis, and prognosis [9–11]. For CCA, studies have confirmed that lncRNA plays a key role in CCA occurrence and progression [12]. For instance, MALAT1 promote CCA cell proliferation and invasion [13], UCA1 affect migration and invasion potential of CCA cells by regulating EMT [14]. Besides, lncRNA such as TUG1 [15], CCAT1 [16], and AFAP1-AS1 [17] could serve as valuable predictive markers for CCA patients prognosis. However, the role and mechanism of lncRNA in the metastasis and recurrence of tumors even in CCA is not completely understood.

In this study, we collected lncRNA expression data and clinical information of CCA patients from two independent database sources to identify and develop a novel lncRNA-based signature panel as an independent predictor, for the prognosis of CCA patients, to guide personalized treatment and hence improve survival. This was achieved by using simple, inexpensive quantitative PCR assays that can be incorporated into the clinical approach. Furthermore, we investigate the possible molecular mechanisms related to this prognostic lncRNA with the occurrence and progress of CCA. We believe this lncRNA-base signature panel offers an effective platform for risk-stratification in CCA patients, which has great implications in the clinical management of patients suffering from this fatal malignancy.

## RESULTS

### Establishment of a five-lncRNA signature predictive model from the TCGA cohort

**Figure 1:**
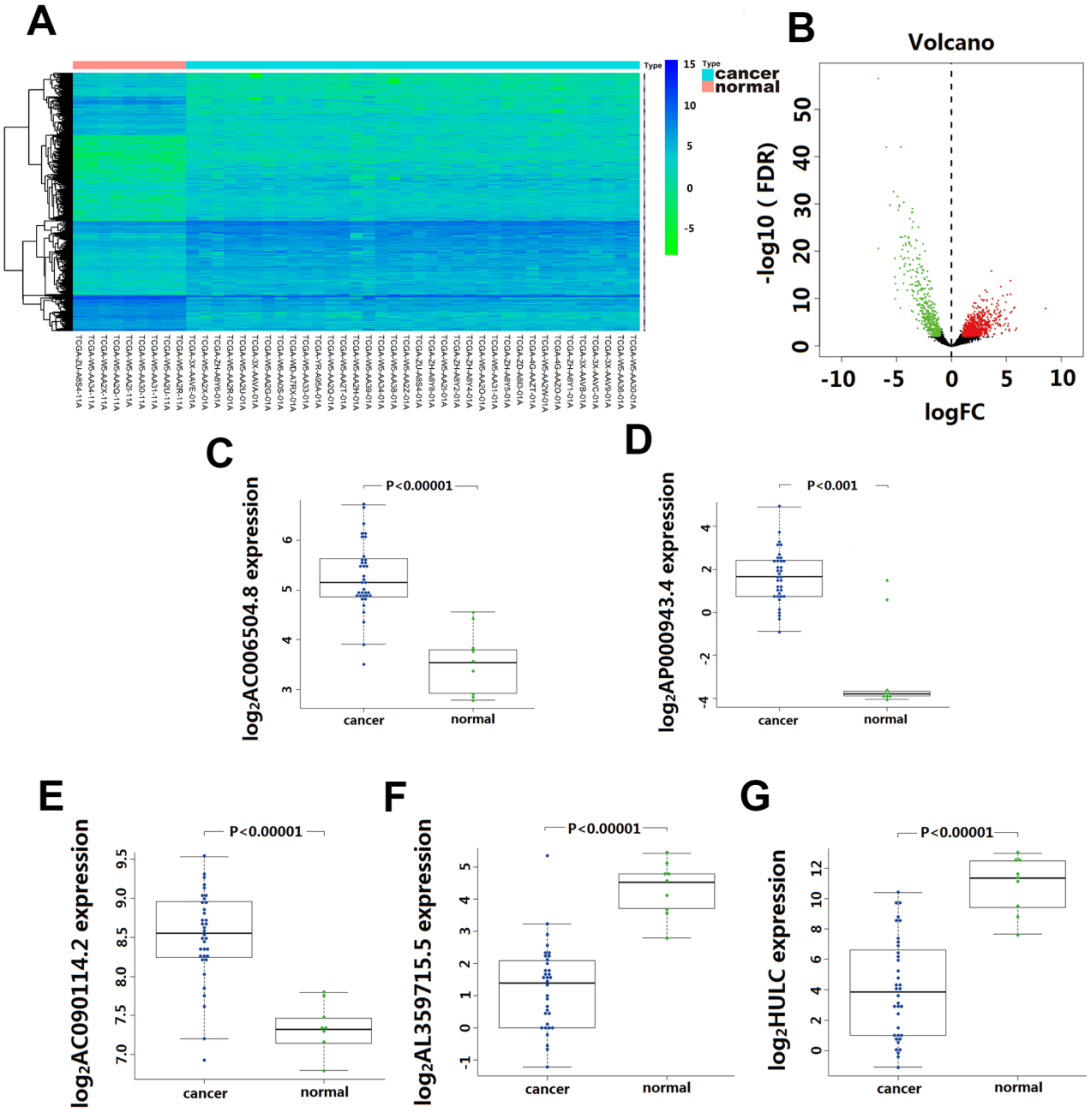
**Differentially expressed lncRNA(DElncRNA) between Cholangiocarcinoma and normal tissues.** (**A**) 1192 DElncRNA were detected based on an unsupervised hierarchical clustering heatmap. 744 DElncRNA expression increased, and 448 DElncRNA expression decreased. The indication from green to red represents the expression level from low to high. (**B**) Volcano plot depicts 1192 DElncRNA (green and red dots) between Cholangiocarcinoma and normal tissues. The X-axis represents log10 fold change; Y-axis represents -log10 of the *p*-value for each lncRNA. LncRNA with fold change >1 or <-1 and FDR < 0.01 were considered to be differentially expressed between tumor and normal tissues. (**C**–**G**) The differentially expressed level of five-lncRNA in 36 CCA tissue and 9 para-carcinoma normal tissue.

Based on the screening criteria, we obtained1192 differentially expressed lncRNA, including 744 up-regulated and 448 down-regulated. Among them, 33 lncRNA showed >4-fold decreased expression including HULC, and 51 lncRNA exhibited >4 fold increased expression (Figure 1A). Unsupervised hierarchical cluster analysis showed that the expression of differentially expressed lncRNA distinguished CCA samples from the normal samples (Figure 1B).

Univariate Cox regression analysis assessing the association between 1192 differentially expressed lncRNA and CCA patients’ overall survival in TCGA cohort, 10 lncRNA (LINC01336; AP000943.4; AC006504.8; AC090114.2; AC004921.1; AC134682.1; AL449106.1; AL359715.5; AC016876.1 and HULC) were selected for further multivariate Cox regression analysis. In this multivariate Cox regression analysis, five lncRNA (AC006504.8, AC090114.2, AP000943.4, AL359715.5, and HULC) were identified as independent predictors for CCA survival outcomes (Table 1). Among them, the expression of AC006504.8, AC090114.2, and AP000943.4 was up-regulated (Figure 1C–1E); expression of AL359715.5 and HULC was down-regulated (Figure 1F, 1G). Similarly, these 5 lncRNAs also showed similar behaviors in paired carcinoma and paracancerous tissues (Supplementary Figure 1). Based on these five lncRNA expression levels and their corresponding coefficient in multivariate Cox regression analysis, a five-lncRNA signature predictive model for CCA patients prognosis was established as follows: Risk Score= (0.6542 × expression level of HULC) + (-1.2388 × expression level of AL359715.5) + (1.3769 × expression level of AC006504.8) + (-3.6697 × expression level of AC090114.2) + (-1.5165 × expression level of AP000943.4).

**Table 1:** Five lncRNA significantly correlated with the overall survival of cholangiocarcinoma.

**Gene name**	**Ensemble ID**	**Chromosome**	HR*	Coefficient*	*P*-value*
HULC	ENSG00000251164.1	chr6:8652137- 8653846	1.9236	3.42	0.00062
AC006504.8	ENSG00000281468.1	chr19:27802838- 27803472	3.9626	2.85	0.00443
AC090114.2	ENSG00000273270.1	chr7:128524016- 128531069	0.0255	-3.28	0.00104
AL359715.5	ENSG00000279022.1	chr6:80440730- 80441172	0.2897	-2.47	0.01366
AP000943.4	ENSG00000280167.1	chr11:94559018- 94559374	0.2195	-3.01	0.00264

Statistics derived from univariate Cox proportional hazards regression analysis in 36 patients in TCGA cohort; HR, Hazard ratio.

### Performance evaluation of the five-lncRNA signature model for CCA prognosis in the discovery cohort

**Figure 2:**
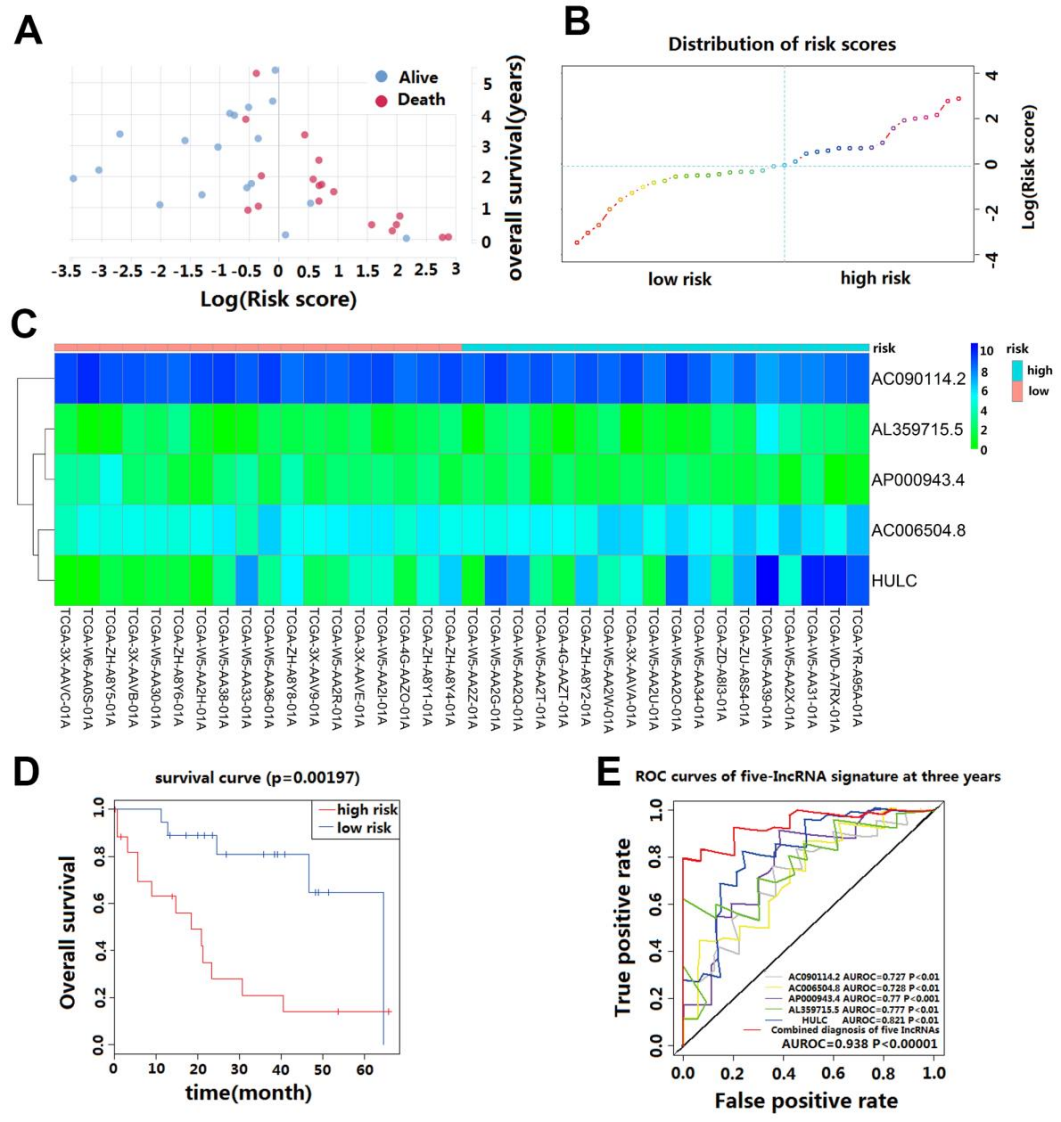
**Prognostic evaluation of the five-lncRNA signature in CCA patients in TCGA.** (**A**) The survival status and duration of CCA patients; (**B**) LncRNA risk score distribution; the blue dashed line indicates dividing the patient into a low-risk group and a high-risk group with a median value as a cut off value. (**C**) Heatmap of five lncRNA expressions in CCA patients. (**D**) KM curves based on OS outcomes for risk cutoffs with a *p*-value of less than 0.01 for the log-rank test. (**E**) Time-dependent ROC curve analysis was predicted by five lncRNA for 3-year survival, and each was performed separately.

We calculated the risk score for each CCA patient in the TCGA cohort based on the five-lncRNA signature model. According to the median of log(Risk Score), -0.0995 was set as the cutoff level to stratify CCA patients into a high-risk or low-risk group(sensitivity is 82.01%, specificity is 86.02%). Finally, 18 patients whose log (Risk Score) >-0.0995 were assigned to the high-risk group, and 18 patients whose log(Risk Score) <-0.0995 were assigned to the low-risk group (Figure 2A–2C). The KM curves show that patients in the high-risk group had a worse prognosis than those in the low-risk group (median OS is 18.5 months vs. 60 months; log-rank *p*=0.002) (Figure 2D). The univariate Cox regression analysis between the lncRNA-based risk score and CCA patient survival score showed that high-risk patients have a worse prognosis (HR=6.760; 95%CI, 1.572-29.068; *p*=0.008). The multivariate Cox regression analysis revealed that high-risk score is an independent predictor for CCA patient survival after adjusting the clinical covariate including recurrence status and residual tumor status (HR=4.879; 95%CI, 1.587-14.996; *p*=0.006) (Table 2).

**Table 2:** Univariate and multivariate Cox regression analysis of cholangiocarcinoma patients in the TCGA cohort.

**Variables**	**Univariate analysis**		**Multivariate analysis**	
	**HR (95% CI)**	***P*-value**	**HR (95% CI)**	***P*-value**
**Age**				
(≥60/<60)	1.600 (0.414-6.177)	0.494		
**Gender**				
(Male/Female)	1.000 (0.269-3.724)	1.000		
**Grade**				
(G3-G4/G1-G2)	1.000 (0.269-3.724)	1.000		
**Stage**				
(III-IV/I-II)	1.923 (0.383-9.646)	0.691		
**T**				
(T2-T3/T1)	1.300 (0.313-5.393)	0.717		
**N**				
(N1/N0)	2.400 (0.339-16.968)	0.625		
**M**				
(M1/M0)	1.731 (0.249-12.011)	0.656		
**Primary pathology residual tumor**				
(R1/R0)	2.000 (0.288-13.910)	0.639		
**Family cancer history**				
(Yes/No)	0.375 (0.085-1.646)	0.188		
**Recurrence**				
(Yes/No)	17.500 (3.312-92.475)	<0.001	7.145 (1.481-37.477)	0.014
**BMI**				
(>24.9/≤24.9)	0.571 (0.130-2.514)	0.571		
**History hepatoma risk factors**				
(Yes/No)	1.257 (0.333-4.742)	0.735		
**Cancer status**				
(With tumor/Tumor free)	24.375 (3.822-155.448)	<0.001	1.845 (0.306-11.142)	0.502
**Postoperative radiotherapy**				
(Yes/No)	0.917 (0.208-4.048)	1.000		
**Primary histological type**				
(Intrahepatic/Other)	1.000 (0.173-5.772)	1.000		
**Five-lncRNA risk score**				
(High/Low)	6.760 (1.572-29.068)	0.008	4.879 (1.587-14.996)	0.006

HR: Hazard Ratio; CI: Confidence Interval.

The AUC value and F1 score were calculated to assess the performance of the five-lncRNA signature model. Among the down-regulated lncRNA, the AUC for AL359715.5 and HULC were 0.777 and 0.821, respectively. The AUC for up-regulated lncRNA AC090114.2, AC006504.8, and AP000943.4 were 0.727, 0.728 and 0.77, respectively. For the whole model, the AUC was 0.938 (Figure 2E), and the F1 score was 0.7222. These results indicate that the five-lncRNA signature model has a high predictive value for CCA prognosis.

### Performance validation of the five-lncRNA signature model in the validation cohort

**Figure 3:**
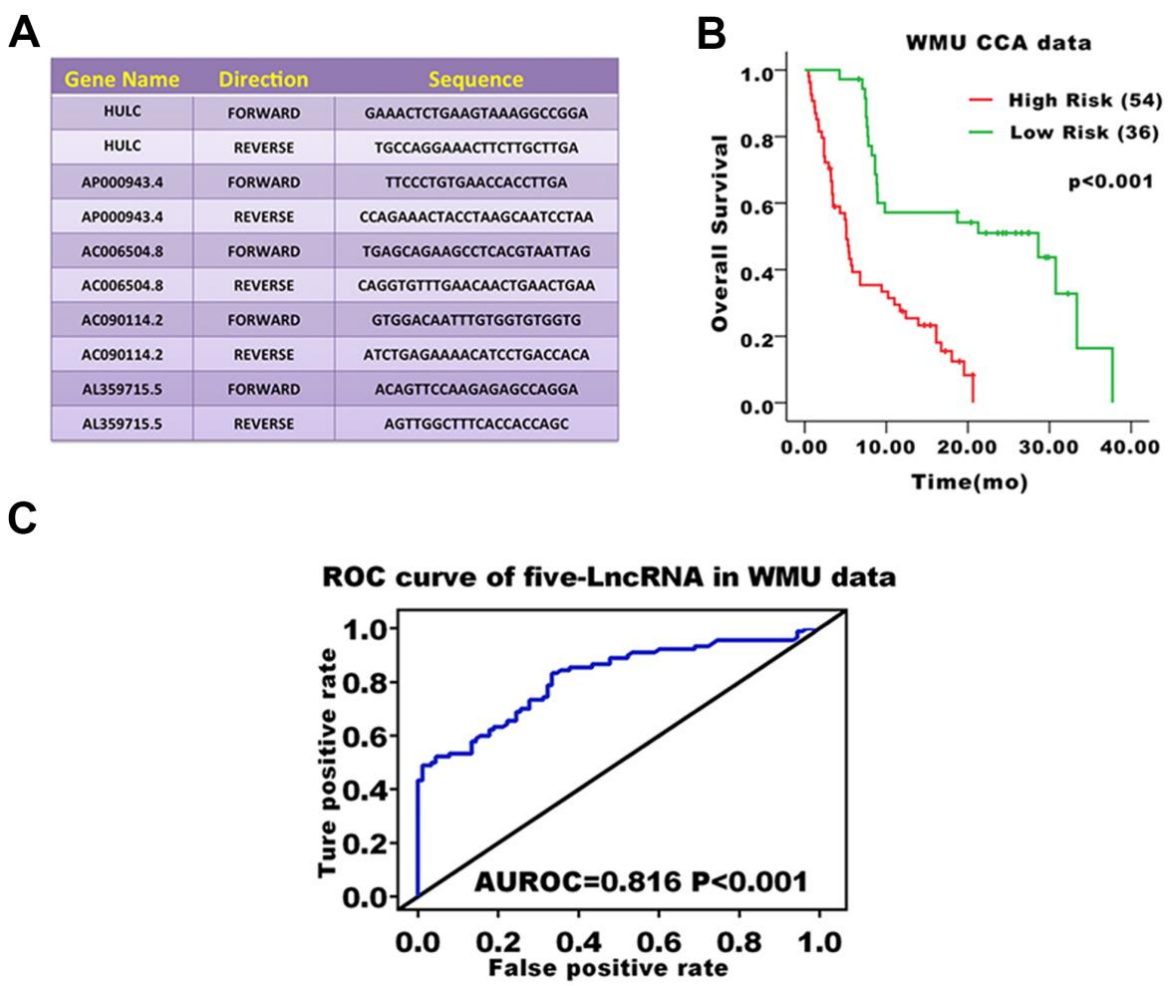
**Prognostic evaluation of the five-lncRNA signature in CCA patients in the WMU cohort.** (**A**) Primer sequence of five-lncRNA markers; (**B**) KM curve analysis of OS validated the prognostic differences between high and low-risk groups in the WMU cohort; (**C**) ROC curve analysis of 3-year survival validated the reliability of five-lncRNA model.

We validated the prediction ability of the five-lncRNA signature model in the WMU cohort to assess the robustness of the model for survival prediction in patients with CCA. Based on the five-lncRNA model and cutoff point derived from the TCGA cohort, patients in the WMU cohort were divided into high-risk group (n=54) and low-risk group(n=36). We compared the KM curve between these two groups and found that the OS of patients in the high-risk group was worse than those in the low-risk group (*p*<0.001, Figure 3B). The AUC value was 0.816 in the WMU cohort (Figure 3C).

### Stratified survival analysis of five-lncRNA model in the TCGA cohort

**Figure 4:**
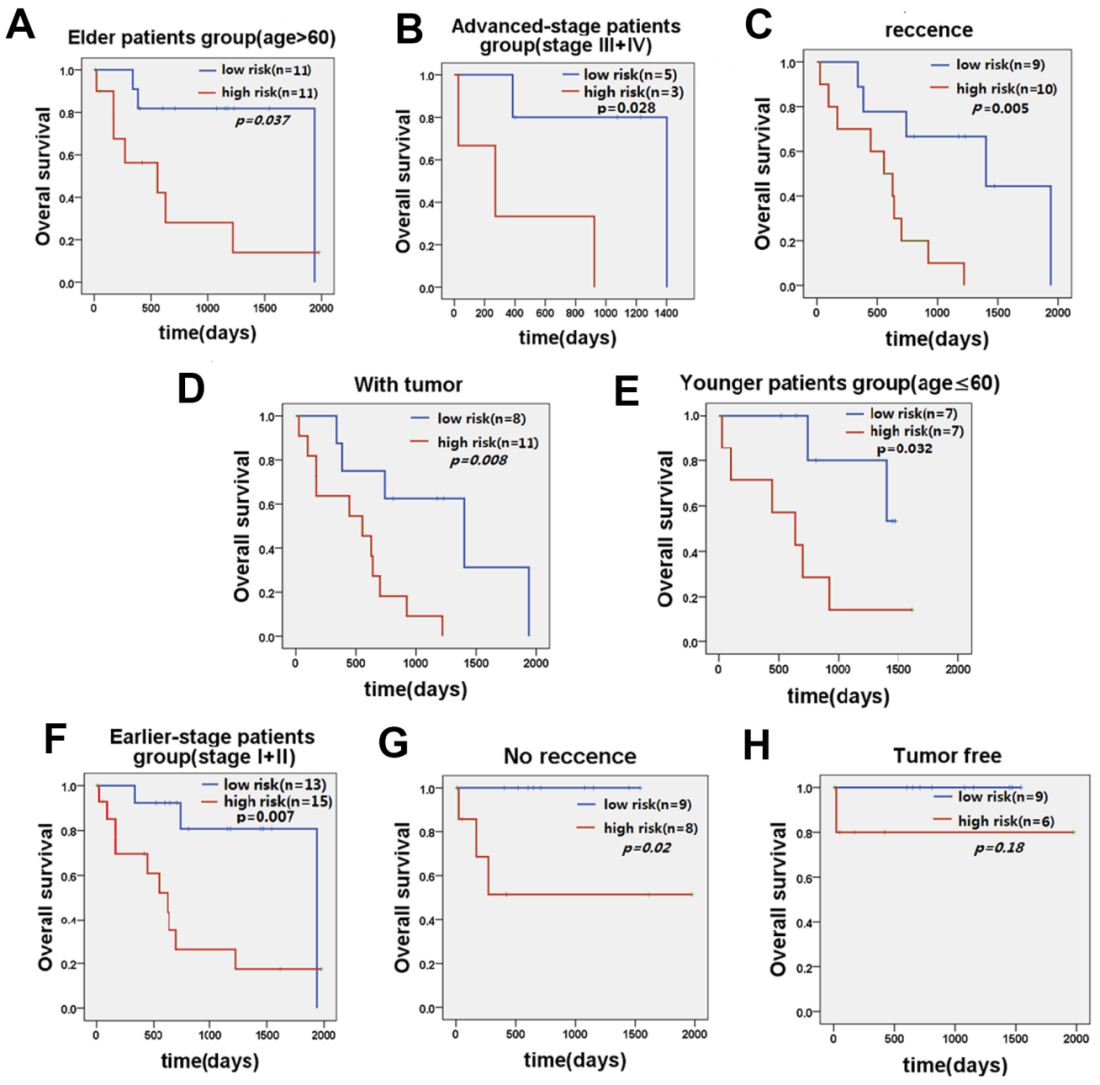
**KM curve of OS of patients stratified by age, stage, recurrence, and current tumor status by five-lncRNA signature.** (**A**) KM curves of the elder patients’ group and (**E**) KM curves in the younger patients’ group; (**B**) KM curves in the advanced-stage patients' group and (**F**) KM curves in the earlier-stage patients' group. (**C**) KM curves in the recurrence patients’ group and (**G**) KM curves in the no recurrence patients’ group. (**D**) KM curves in the with tumor patients’ group and (**H**) KM curves in the tumor-free patients’ group.

Further stratified analysis showed that the five-lncRNA signature model could accurately distinguish the prognosis between the high-risk group and the low-risk group in young patients <60 years (n=14, *p*=0.032, Figure 4E) and older patients ≥60 years (n=22, *p*=0.037, Figure 4A). Similarly, stratifying the patients based on the stage of disease, revealed that the five-lncRNA signature model has good discriminatory ability for earlier-stage patients (n=28, *p*=0.007, Figure 4F) and advanced-stage patients (n=8, *p*=0.028, Figure 4B). For patients with or without recurrence, the five-lncRNA model could divide patients into the high-risk or low-risk groups in those with recurrence (n=19, *p*=0.005, Figure 4C) and without recurrence (n=17, *p*=0.02, Figure 4G). Moreover, the five-lncRNA signature model could separate the high-risk group and low-risk group for patients with tumors (n=19, *p*=0.008, Figure 4D) and those who were tumor-free (n=15, *p*=0.18, Figure 4H). Multivariate Cox regression analysis combined with stratification analysis showed that there was no significant difference in OS between the high-risk and low-risk groups with five-LncRNA markers in tumor-free patients, and it this suggests that patients in the early stages of tumor development may benefit significantly from these prognostic biomarkers.

### Identifying the functions of the five-lncRNA signature model

Co-expression analysis showed significant co-expression of 1429 DPCGs, 1440 DPCGs, 300 DPCGs, 495 DPCGs, and 552 PCGs with HULC, AL359715.5, AP000943.4, AC006504.8, AC090114.2, respectively. Functional enrichment analysis indicated that 72 GO biological processes (BP) terms, 21 GO cellular components (CC) terms, and 35 GO molecular functions (MF) terms were enriched for HULC-related DPCGs. Biological processes were mainly involved in the oxidation-reduction process, xenobiotic metabolic process, metabolic process; cellular components were mainly involved in extracellular exosome, mitochondrial matrix, blood microparticle; molecular functions were mainly involved in oxidoreductase activity, electron carrier activity, monooxygenase activity (Supplementary Figure 2A, 2C). There was significant enrichment of 60 KEGG pathways in HULC-associated DPCGs, including leucine, isoleucine and valine degradation, complement and coagulation cascades, fatty acid degradation, carbon metabolism and chemical carcinogenesis (Supplementary Figure 2B, 2C).

47 GO BP terms, 11 GO CC terms, and 33 GO MF terms were enriched for AL359715.5-related DPCGs, whose biological processes were mainly associated with drug metabolic process, lipid metabolic process, lipoprotein metabolic process; cellular components were mainly associated with organelle membrane, mitochondrion, peroxisome; molecular functions were mainly associated with iron ion binding, heme binding, cholesterol transporter activity (Supplementary Figure 3A, 3C).

56 KEGG pathways were enriched for AL359715.5-related DPCGs, which were primarily linked to Drug metabolism - cytochrome P450, Metabolism of xenobiotics by cytochrome P450, Retinol metabolism, Peroxisome, and Cholesterol metabolism (Supplementary Figure 3B, 3C).

16 GO BP terms, 18 GO CC terms and 4 GO MF terms were enriched for AC006504.8-related DPCGs, whose biological processes were mainly involved in cell division, sister chromatid cohesion, mitotic nuclear division; cellular components were mainly involved in condensed chromosome kinetochore, midbody, nucleoplasm; molecular functions were mainly involved in protein binding, ATP binding, and cadherin binding involved in cell-cell adhesion (Supplementary Figure 4A, 4C). It was a significant enrichment of 7 KEGG pathways in AC006504.8-related DPCGs, including DNA replication, cell cycle, the p53 signaling pathway, Fanconi anemia pathway and progesterone-mediated oocyte maturation (Supplementary Figure 4B, 4C).

7 GO BP terms, 2 GO CC terms and 1 GO MF terms were raised in AC090114.2-associated DPCGs, whose biological processes were mainly related to sister chromatid cohesion, DNA replication initiation, G1/S transition of mitotic cell cycle; cellular components were mainly associated with cytosol and MCM complex; molecular functions were mainly associated with protein binding (Supplementary Figure 5A, 5C). 4 KEGG pathways were raised in AC090114.2-related DPCGs, which were mainly related to DNA replication, cell cycle, cellular senescence and oocyte meiosis (Supplementary Figure 5B, 5C).

1 GO CC terms and 1 GO MF terms could be found for DPCGs related to AP000943.4, whose cellular components were associated with cytoskeleton; molecular functions were associated with extracellular matrix organization and involved a KEGG pathway for Human papillomavirus infection (Data not shown).

### Functional evaluation of common DPCGs for five-lncRNA signature model

**Figure 5:**
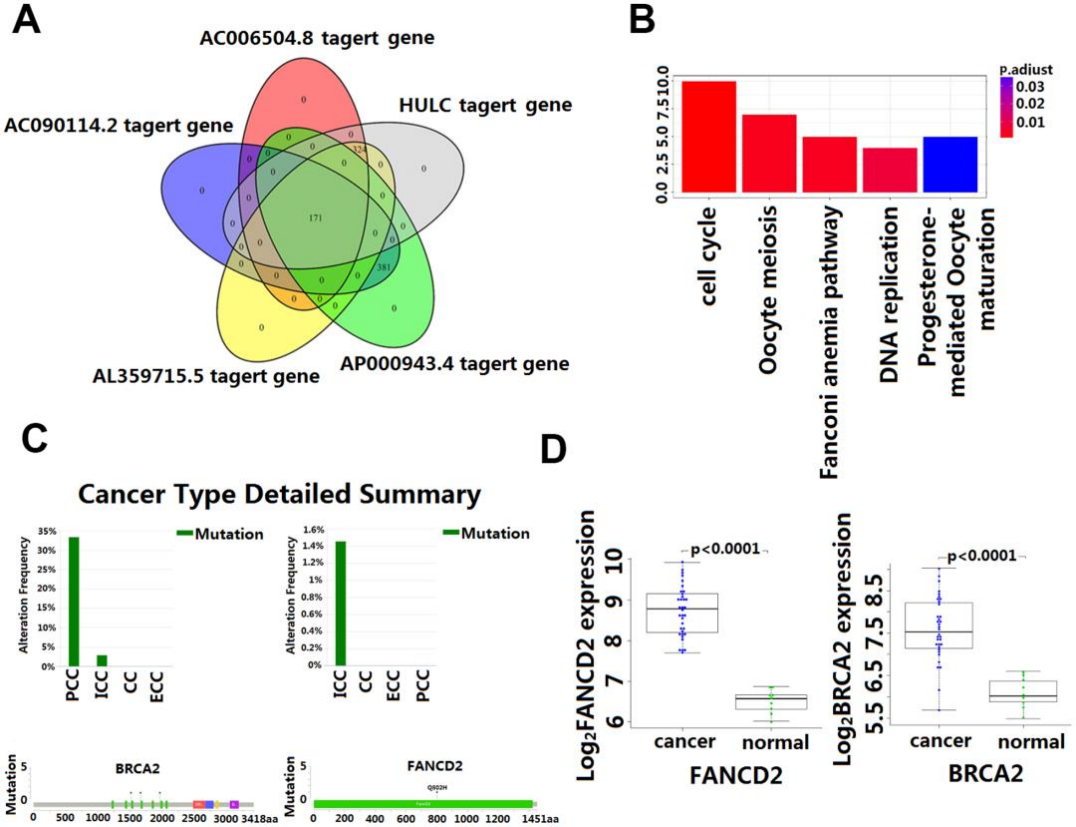
**Enrichment and analysis of five lncRNA in the presence of common DPCGs.** (**A**) Venn diagram showing 171 common DPCGs of the five-lncRNA. (**B**) The KEGG pathways were significantly associated with the enrichment of 171 common protein-coding genes co-expressed with five-lncRNA. The ordinate is the number of DPCGs that is enriched to the target gene. (**C**) Mutation of FANCD1 and FADCD2 genes in cholangiocarcinoma (from the cbioportal database http://www.cbioportal.org/). (**D**) Expression of FANCD1 and FADCD2 genes in cholangiocarcinoma.

The intersection of the DPCGs corresponding to the five-LncRNA signature model showed that 171 DPCGs were shared by this five-LncRNA signature (Figure 5A). The common KEGG pathway is cell cycle, DNA replication, oocyte meiosis, Fanconi anemia pathway, and progesterone-mediated oocyte maturation (Figure 5B and Supplementary Table 1).

### GSEA between the high-risk group and low-risk group

**Figure 6:**
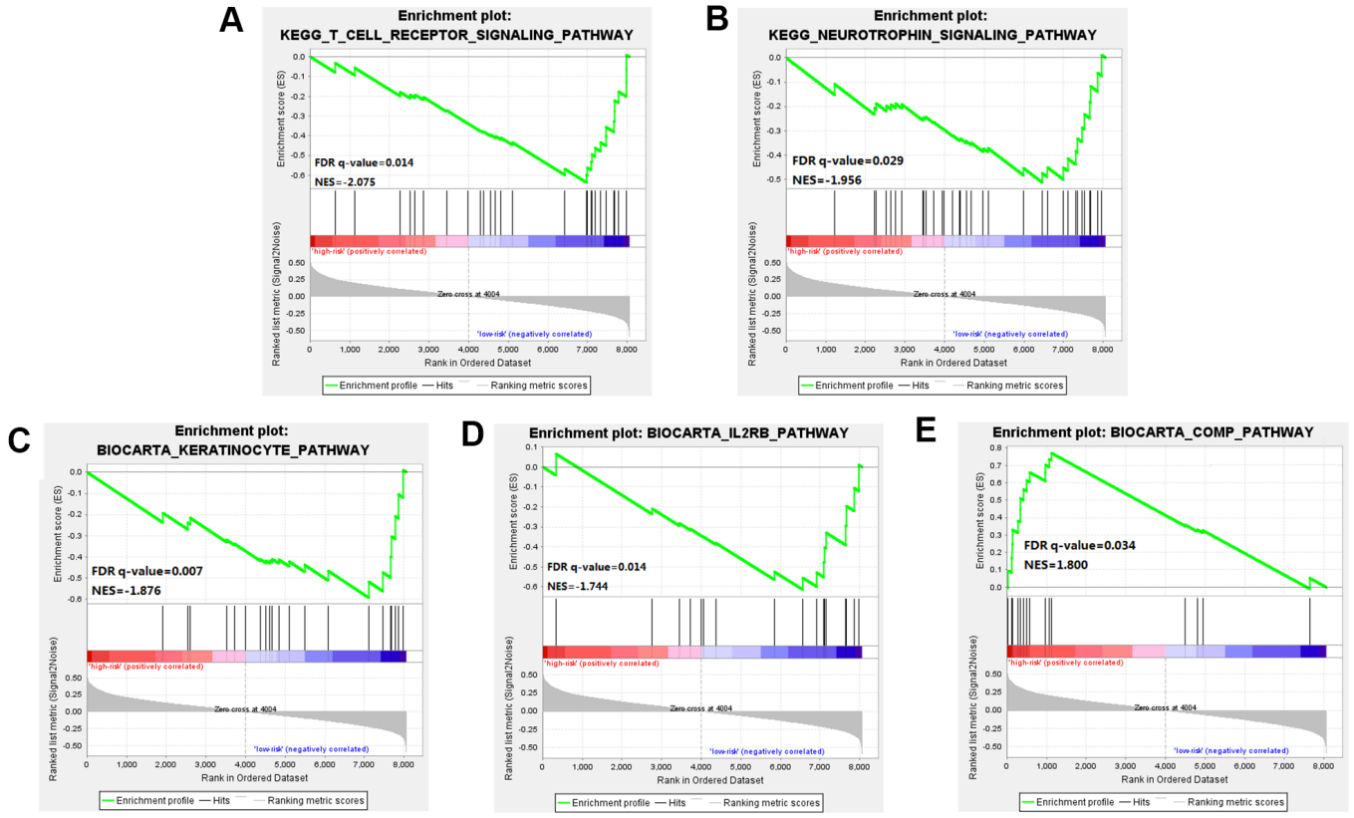
**Gene Set Enrichment Analysis (GSEA) was performed between the high risk score group and the low-risk score group.** (**A**–**D**) Pathways including IL-2 Receptor Beta Chain in T cell Activation, Keratinocyte Differentiation, T cell receptor pathway, and Neurotrophin signaling pathway were enriched in the low-risk group. (**E**) The results showed significant enrichment of markers including the "complement pathway" in the high-risk group.

Through GSEA analysis, we clarified the significant difference in survival between the high-risk and low-risk groups. The results showed significant enrichment of markers including the "complement pathway" in the high-risk group. Pathways including IL-2 Receptor Beta Chain in T cell Activation, Keratinocyte Differentiation, T cell receptor pathway, and Neurotrophin signaling pathway were enriched in the low-risk group [18], many of these being closely related to the occurrence and development of cancer [19] (Figure 6 and Supplementary Table 2).

## DISCUSSION

Currently, the molecular genotype for a variety of tumors (breast cancer, gastric cancer, and colorectal cancer) has been applied in a clinical setting. Some molecular genotypes are not only used to predict the prognosis but also to select the best therapy target [20]. The comprehensive study of the mechanism has led to the discovery of many kinds of targeted drugs used in the treatment of these diseases [21]. However, for CCA, there are relatively few studies on prognostic molecular markers. Therefore, establishing a molecular prediction model in CCA for guiding personalized treatment and predicting prognosis is particularly urgent. In this study, we established a prediction model based on five lncRNA for the prognosis of CCA and validate its reliability in an independent clinical center biobank. The molecular mechanism of these five lncRNA was further explored by the signal pathway analysis.

There is growing evidence that lncRNA plays a key role in transcription and post-transcriptional regulation of gene expression [22–24] as well as in different cells and developmental processes [25–27]. Experimental evidence indicates that abnormal expression of lncRNA is relative to the onset of various diseases including gastric cancer, breast cancer, HCC, lung cancer, and CCA [28–30]. Recent reports indicate that oxidative stress up-regulates the dysfunction of lncRNA H19 and HULC, and then modulates CCA cell migration and invasion through ceRNA targeting IL-6 and CXCR4 [31]. Similarly, the lncRNA CPS1-IT1 is up-regulated in intrahepatic CCA. Conversely, knockdown of CPS1 and/or CPS1-IT1 reduced the proliferation and increased apoptosis of ICC-9810 cells [30]. By comparing the expression of AFAP1-AS1 in CCA tissues and paired adjacent tissues and analyzing the relationship between AFAP1-AS1 expression and the clinical features of CCA, it was discovered that AFAP1-AS1 is significantly associated with the malignant degree and poor prognosis of CCA. Studies have shown that knockdown AFAP1-AS1 inhibits tumor growth *in vivo* and inhibits cell proliferation and invasion *in vitro* [32]. Other studies have found that certain lncRNA play a critical role in the metastasis and malignant progression of CCA. It has been reported that some lncRNA increased in the tissues of patients with advanced CCA and lymph node metastasis, and through inhibition and overexpression in lncRNA experiments, it was found that this overexpression of certain lncRNA may promote the growth and metastasis of CCA through some miRNA (miRNA-200c, miR-296-5p, et al.) [31]. Another study has found that lncRNA-DANCR can bind to EZH2 and regulate histone methylation FBP1 promoter expression, which regulates the growth and migration of CCA cells [33].

Although the study of the lncRNA function has attracted more and more attention and a great number of lncRNA have been identified in the human genome, the function of most lncRNA has not been fully revealed. Functional annotation of the gene encoding the lncRNA-associated co-expressed protein is a viable method for finding the biological characteristics of lncRNA [31]. By extension, annotation of LncRNA function through co-expressed genes was reported to be effective [34]. In this study, GO and KEGG enrichment analysis was used to identify co-expressed mRNAs of the five lncRNA to speculate on the functions of the predictive lncRNA. Our data revealed that the HULC and AL359715.5 participated in a number of biological processes that were most relevant to the cholesterol and fatty acid metabolism which is reported to be responsible for the growth and accelerated development of CCA [34, 35]. Also, of interest is the identification of the complement and coagulation cascades that are involved in many physiological and pathological processes, including those in the inflammatory process which, once dysregulated become an important factor in tumorigenesis [36]. In this study, we found that AC006504.8 was enriched in the p53 signaling pathway. The molecular epidemiological analysis revealed that p53 is mutated in almost all kinds of tumors, and approximately 5% of patients with colorectal cancer, lung cancer, melanoma, sarcoma, head and neck cancer, leukemia, esophageal cancer, ovarian cancer, testicular cancer, and cervical cancer have been found to have p53 mutations [37, 38]. Of significance to this study is the amount of research that has indicated p53 inactivation plays a key role in the occurrence and development of CCA [39]. The mechanisms by which AC006504.8 is involved in CCA are probably related to cell cycle and DNA replication. The 171 DPCGs intersected by the five-lncRNA signature were enriched in the function of the Fanconi anemia (FA) pathway. Fanconi anemia is a recessive genetic disorder characterized by congenital malformation, bone marrow failure, and high susceptibility to cancers [36, 40]. It is a cancer susceptibility gene involved in the repairing of genomic damage and maintaining genomic stability [41]. Recent evidence indicates that genetic instability is a key factor in the metastasis and recurrence of malignant tumors. Many studies have shown that mutations and abnormal expression of the FANCD1 and FANCD2, two major genes in the Fanconi anemia pathway, are significantly associated with poor prognosis of CCA [42]. Our study also showed that FANCD1 and FANCD2 mutated to different degrees in CCA (Figure 5C), and their expression in CCA and matched para-carcinoma tissues was also significantly different (Figure 5D). These results would seem to suggest that the predictive five-lncRNA may mediate the development and progression of CCA via DPCG interactions in biological processes related to cancer. However, more experimental studies are needed to further explain the potential roles of these lncRNA in CCA. To our knowledge, four out of the five lncRNA biomarker functions have never been reported. Therefore, we postulate that further investigation of the function of the lncRNA will contribute to early diagnosis and provide a clinical basis for the development of new prognostic factors in CCA.

In summary, we systematically studied the lncRNA expression profiles of CCA patients and their corresponding clinical information and found five-lncRNA (HULC, AP000943.4, AC006504.8, AC090114.2, AL359715.5) signature showing the risk scoring model in this study was an excellent way to classify patients with different survival outcomes. Also, the five lncRNA molecular biomarkers performed very well in predicting 3-year survival in patients with CCA, which could be an independent predictor of survival prognosis, and which could provide novel insights into the molecular mechanisms of CCA tumorigenesis and development. However, this study has some limitations. Firstly, our five-lncRNA signature model was only tested and validated in the TCGA and WMU cohort. If possible, it should be validated in other independent cohorts. Secondly, our research only investigated the biological function of predictive lncRNA by computational methods. It should be supplemented with *in vitro* cell research and animal experiments *in vivo*. Combining these data will help to unravel the mechanism of lncRNA involved in CCA tumorigenesis.

## MATERIALS AND METHODS

### Screening differentially expressed lncRNA in CCA patients in the discovery cohort

Pre-processed level 3 RNA sequencing count data and relative clinical information for CCA patients were obtained from the Genomic Data Commons Data Portal database (TCGA, https://portal.gdc.cancer.gov/projects/TCGA-CHOL) [43]. Ultimately, 60,483 RNA-ENSG_ID expression profiles in 36 CCA patients were included for further analysis as the discovery cohort (TCGA cohort).

The Gencode.v27.long_noncoding_RNAs.gtf compressed file was downloaded from the GENCODE database (release 27) (http://www.gencodegenes.org/) and the transformed data (antisense, lincRNA, and sense_intronic) was determined as lncRNA [44]. It was then filtered by removing the exon-expressing lncRNA from any known coding gene by GENCODE-based gene annotation. A total of 59,264 lncRNA-ENSG_IDs, and RNA-ENSG_ID and lncRNA-ENSG_ID were intersected, 13,126 lncRNA-Gene_names were obtained (Supplementary Figure 6). Samples in which the lncRNA with RPKM expression value is 0 more than 20% were removed. Finally, 3651 lncRNA were keptfor further analysis. Furthermore, we downloaded Homo_sapiens.GRCh38.91.chr.gtf zip file from Ensembl genome browser 91 (ftp://ftp.ensembl.org/pub/release-91/gtf/homo_sapiens) to obtain 57,000 RNA-Gene_symbol expression profiles, then used "mygene" package in R to find the RNA with human entrezgene symbol (GeneID) in the 57,000 RNAs.

Differentially expressed genes (DEGs) between 36 CCA tissues (30 iCCA tissues, 6 pCCA/dCCA tissues) and 9 normal tissues with the |log_2_ fold change (log_2_ FC)| ≥1 and false discovery rate(FDR) <0.01 were considered as selection criteria for subsequent analysis. At the same time, differentially expressed analysis was performed on the RNAs identified with GeneID.

### Establishment and performance evaluation of a lncRNA-based prediction model in the discovery cohort

Firstly, we assessed the association between differentially expressed lncRNA and the overall survival (OS) of CCA patients in the TCGA cohort by univariate Cox proportional hazards regression analysis. LncRNA that were statistically significant were included in further multivariate Cox regression analysis. Then, using the multivariate Cox regression analysis, we identified independent lncRNA predictors. Furthermore, the corresponding coefficients of each lncRNA in the Cox regression model were obtained to calculate the risk score [45]. Finally, a lncRNA-based prediction model to evaluate the CCA patient survival outcomes was established as below:

$$\text{LncRNA-based Risk Score}=\sum_{i=1}^{N}\left(Coei\times EVi\right)$$

In this formula, N represents the number of prognostic lncRNA, Coe_i_ is the coefficient of the lncRNA_i_ in the multivariate Cox regression analysis, EV_i_ represents the expression level of the lncRNA_i_.

We used the lncRNA-based model to calculate the risk score for each CCA patients in the TCGA cohort. Setting the median value of log (Risk Score) as a threshold, CCA patients were divided into high-risk and low-risk groups and the difference of OS between these two groups was compared. Then univariate and multivariate Cox regression analysis was performed to determine whether the lncRNA signature was an independent predictor variable of other clinicopathologic features for survival outcomes. Further stratification analysis was conducted on clinicopathologic characteristics, which were statistically significant in a multivariate Cox regression model to determine the lncRNA signature model’s predictive capacity within the same clinical features. We calculated the area under the time-dependent receiver operating characteristic (ROC) curve (AUC) within a 3-year survival period to evaluate the sensitivity and specificity of the lncRNA model to predict survival outcomes.

### Verification of lncRNA signature model for survival prediction in the validation cohort

We performed validation of discovered lncRNA in fresh frozen tissues from 90 CCA patients who underwent surgery between November 2012 and December 2015 at the First Affiliated Hospital of Wenzhou Medical University (WMU cohort). The patient inclusion criteria were as follows: (1) pathological diagnosed with primary CCA; (2) had completed clinicopathological and follow-up monitoring; (3) had no anti-tumor treatment before this surgical resection. Exclusion criteria were as follows: (1) previous radiofrequency or other anti-tumor treatment before surgery; (2) patients who were lost to follow-up after surgery. The study was approved by the institutional review boards of First Affiliated Hospital of Wenzhou Medical University and written informed consent was obtained from all patients. Patients demographics and clinicopathological characteristics are shown in Supplementary Table 3.

The lncRNA expression of primary CCA tumor fresh frozen samples was assessed by real-time quantitative PCR. RNAeasy mini kit (Qiagen, CA, USA) was used to extract the total RNA. High-Capacity cDNA Reverse Transcription kit from Applied Biosystems (Grand Island, NY, USA) was used to synthesize cDNA from 2 μg of total RNA. Semi-quantitative detection of mRNA was performed using an ABI 7300 RT-PCR system. Relative quantification of mRNA levels was performed with 18S ribosomal RNA as an internal reference gene and data from the ΔΔCt method. The statistical mean and standard error were determined by the ΔCt value. All data were independently inputed three times. The primer sequences used in the present study are shown in Figure 4A.

Through the same lncRNA signature model and cutoff level derived from the discovery cohort, patients in the WMU cohort were divided into high-risk and low-risk groups. Then, we investigated the performance of the lncRNA signature model in the WMU cohort.

### Co-expression and functional enrichment analysis

The Spearman correlation coefficient was calculated between lncRNA expression level and the differentially expressed protein-coding genes (DPCGs). The DPCGs with Spearman correlation coefficient greater than 0.50 were considered to be lncRNA-related DPCGs. Gene Ontology (GO), Cell Component (CC), Biological Process (BP), Molecular Function (MF), Cell Component (CC) and Kyoto Encyclopedia Gene and Genome (KEGG) pathway enrichment assays were performed using the Database for Annotation, Visualization, and Integrated Discovery (DAVID, version 6.8, https://david.ncifcrf.gov/) [46]. Gene Set Enrichment Analysis (GSEA 3.0) analyses were carried out to elucidate the survival difference between high-risk and low-risk groups. GSEA analyses were implemented with java software GSEA (http://software.broadinstitute.org/gsea/index.jsp) [18]. GO (BP, MF, CC) terms, KEGG pathways, and GSEA analyses with adjusted *P* value or a false discovery rate (FDR) less than 0.05 were considered statistically significant.

### Statistical analysis

The continuous variables were expressed as mean ± standard deviation or median (quartile range), and categorical variables were presented as frequencies (percentages). A Chi-square test was used to compare the differences between independent groups. Cox proportional hazard regression analysis was conducted to evaluate the association of lncRNA signature in predicting overall survival in CCA patients. Kaplan-Meier (KM) analysis was used to determine survival outcomes. The median values were used as a cut-off level to plot the KM curves, and the log-rank test was performed to evaluate the statistical significance. The results of the stepwise multivariate Cox regression analysis of the AIC (Akaike Information Criterion, assessing the goodness of fit of a statistical model) test yielded a predictive model with optimal interpretation and information effectiveness. A linear correlation model was performed to evaluate the relationships between the variables and the Pearson correlation coefficient or Spearman rank correlation coefficient was used to present the result. Unless otherwise indicated, all statistical tests were two-sided and a *p*-value<0.05 was considered as statistically significant. All data analysis was performed with R (version 3.3.3; http://www.r-project.org/). The differential expression of the lncRNA profile was estimated by the R “edgeR” package. Unsupervised hierarchical clustering analysis was accomplished by the R “pheatmap” package and represented as a volcano plot. KM survival analysis and Cox proportional hazard regression analysis was performed by the R “survival” package. The AUC value was calculated by the R “Survival ROC” package. The R “clusterProfiler”, “pathview” and “venn” package were used to find the common DPCGs of lncRNAs for KEGG pathways.

### Data sharing and data accessibility

The data used to support the findings of this study are available from the corresponding author upon request.

## Supplementary Material

Supplementary Figures

Supplementary Tables
