# Supplementary material for: A novel five-lncRNA signature panel improves high-risk survival prediction in patients with cholangiocarcinoma
Source: Aging (Albany NY). 2021 Jan 20;13(2):2959–81. doi: 10.18632/aging.202446 (PMC7880389; doi:10.18632/aging.202446)
Supplement: Supplementary Figures [file aging-13-202446-s001.pdf]

SUPPLEMENTARY FIGURES

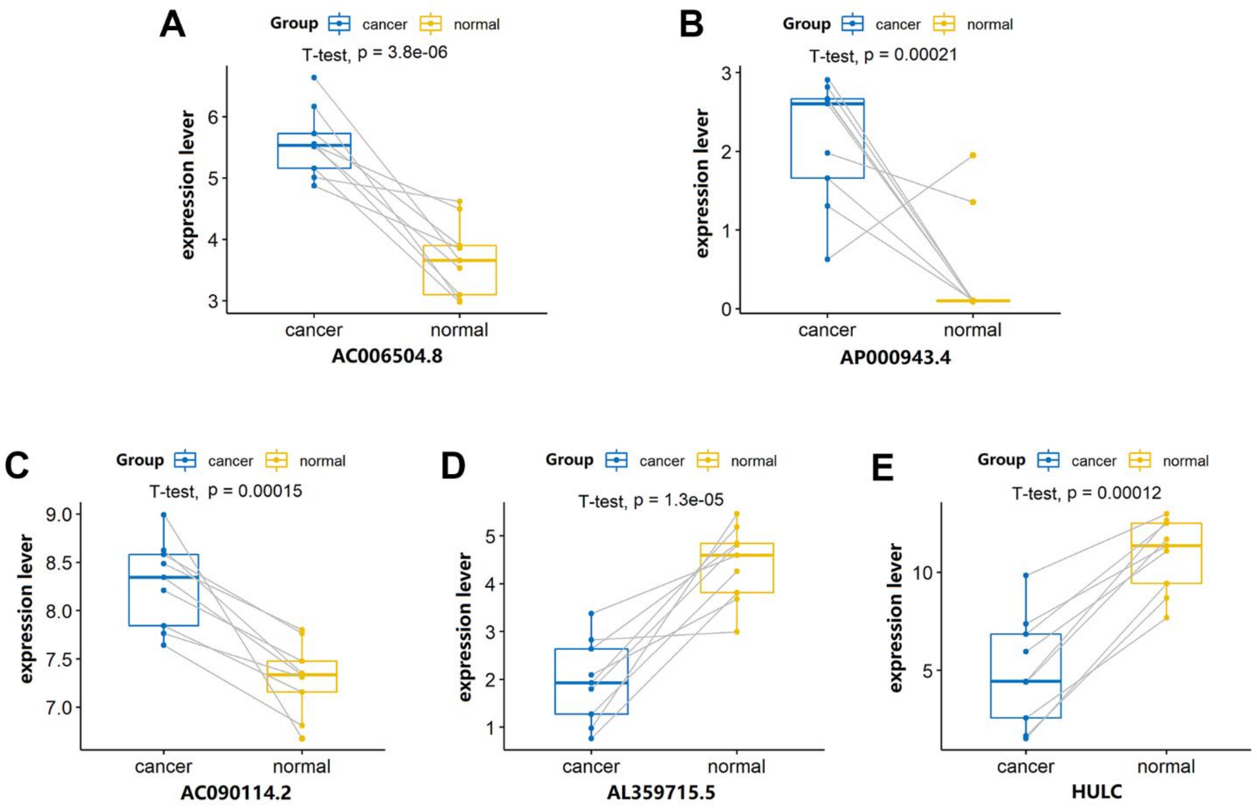

Supplementary Figure 1. The differentially expressed level of five-lncRNA in paired carcinoma and paracancerous tissues. (**A**–**C**) AC006504.8, AP000943.4 and AC090114.2 expressed higher in cancer tissues. (**D**, **E**) AL359715.5 and HULC expressed lower in paracancerous tissues.

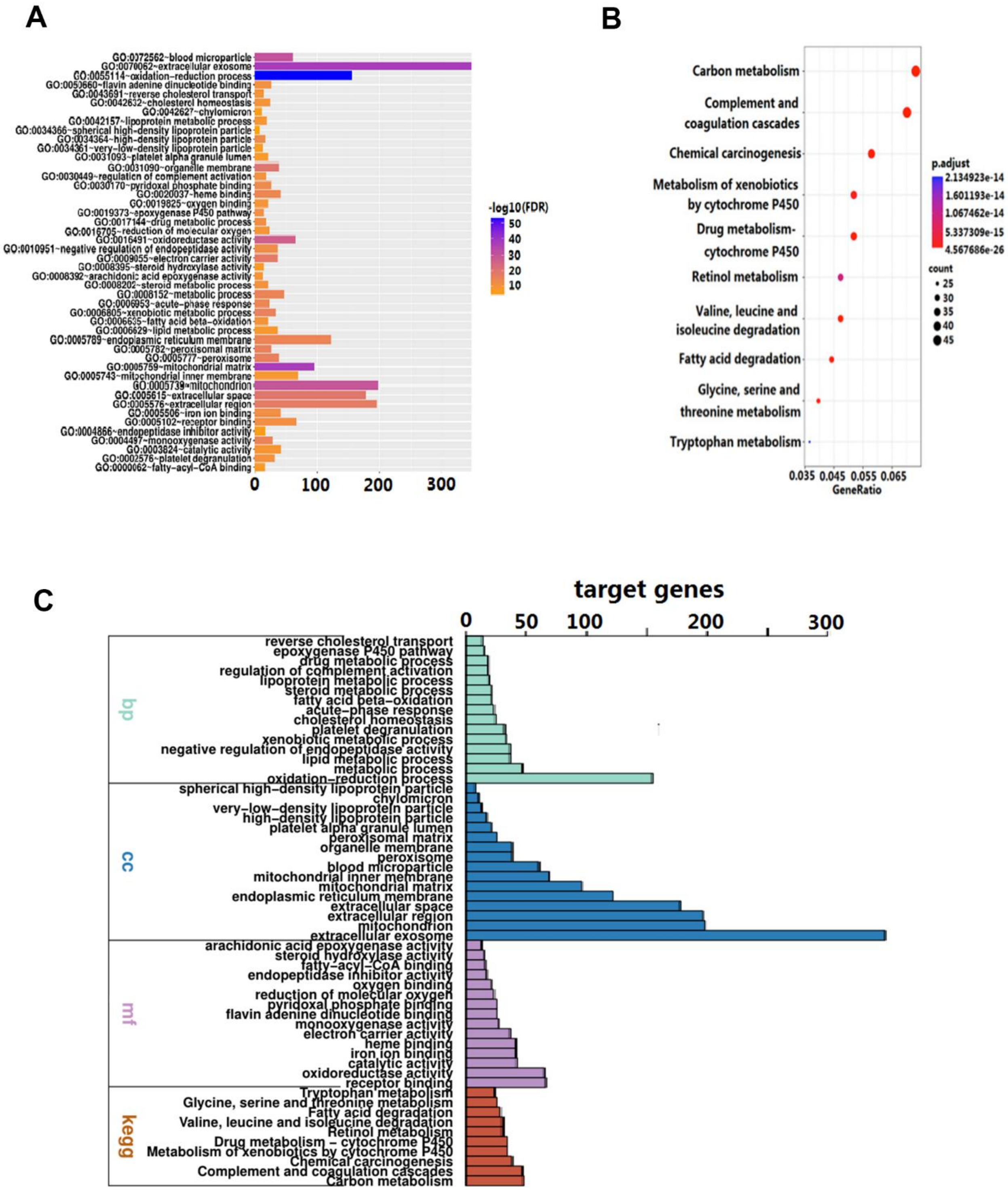

Supplementary Figure 2. Protein-coding genes co-expressed with HULC lncRNA enriched by significant KEGG pathways and GO-based functional enrichment analysis. (**A**) The top 15 biological processes (BP), top 15 cellular components (CC) and top 15 molecular functions (MF) enriched among 1429 DPCG targets of HULC lncRNA. (**B**) Top 10 KEGG pathways for HULC lncRNA-related DPCGs. (**C**) Number of target genes enriched (the horizontal axis represented the corresponding -Log10 (FDR) of different paths and the vertical axis denoted the path name; bp: Biology Process; cc: Cellular Components; mf: Molecular Function).

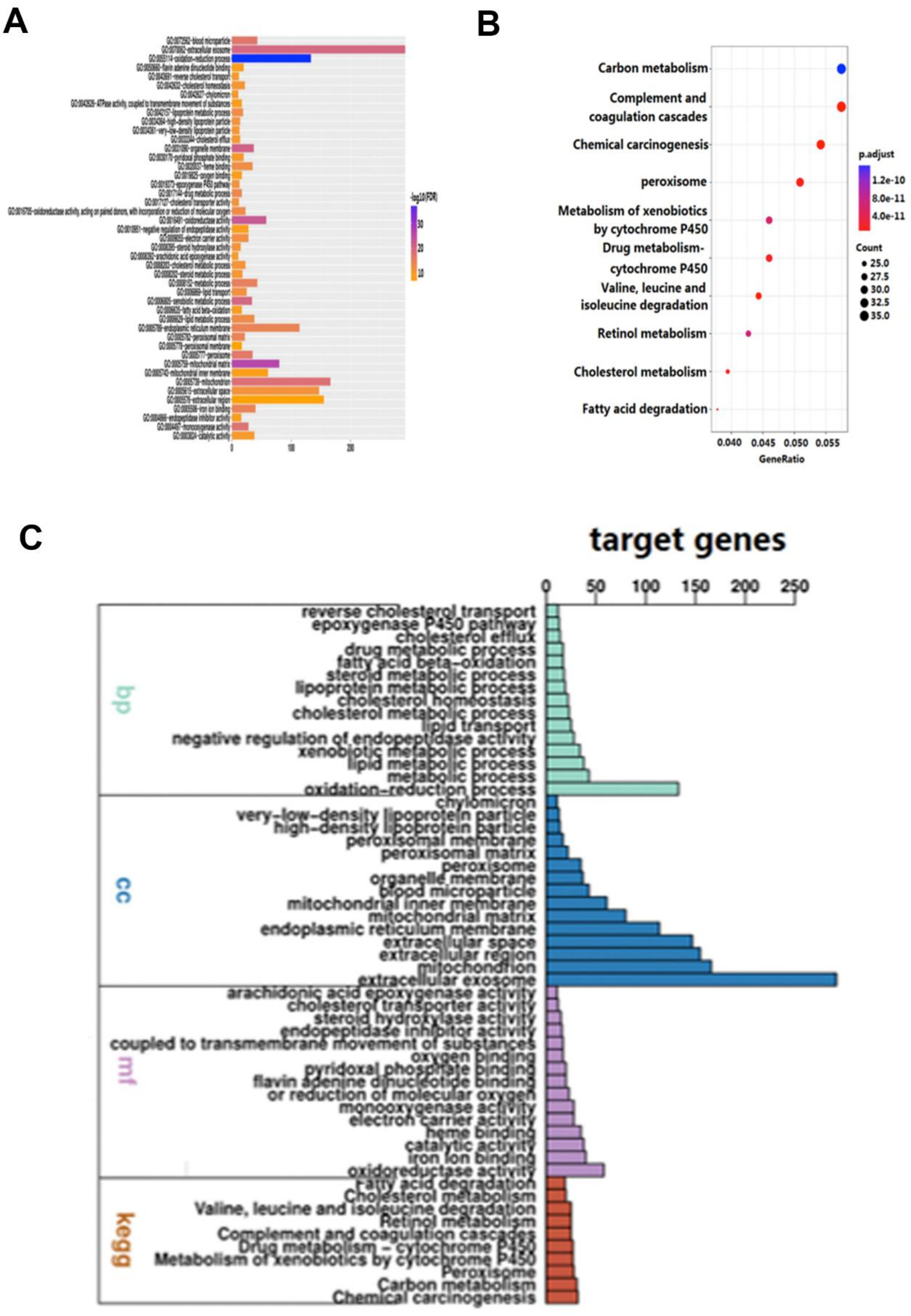

Supplementary Figure 3. Protein-coding genes co-expressed with AL359715.5 lncRNA enriched by significant KEGG pathways and GO-based functional enrichment analysis. (**A**) The top 15 biological processes (BP), top 15 cellular components (CC) and top 15 molecular functions (MF) enriched among 1440 DPCG targets of AL359715.5 lncRNA. (**B**) Top 10 KEGG pathways for AL359715.5 lncRNA-related DPCGs. (**C**) Number of target genes enriched (the horizontal axis represented the corresponding -Log10 (FDR) of different paths and the vertical axis denoted the path name; bp: Biology Process; cc: Cellular Components; mf: Molecular Function).

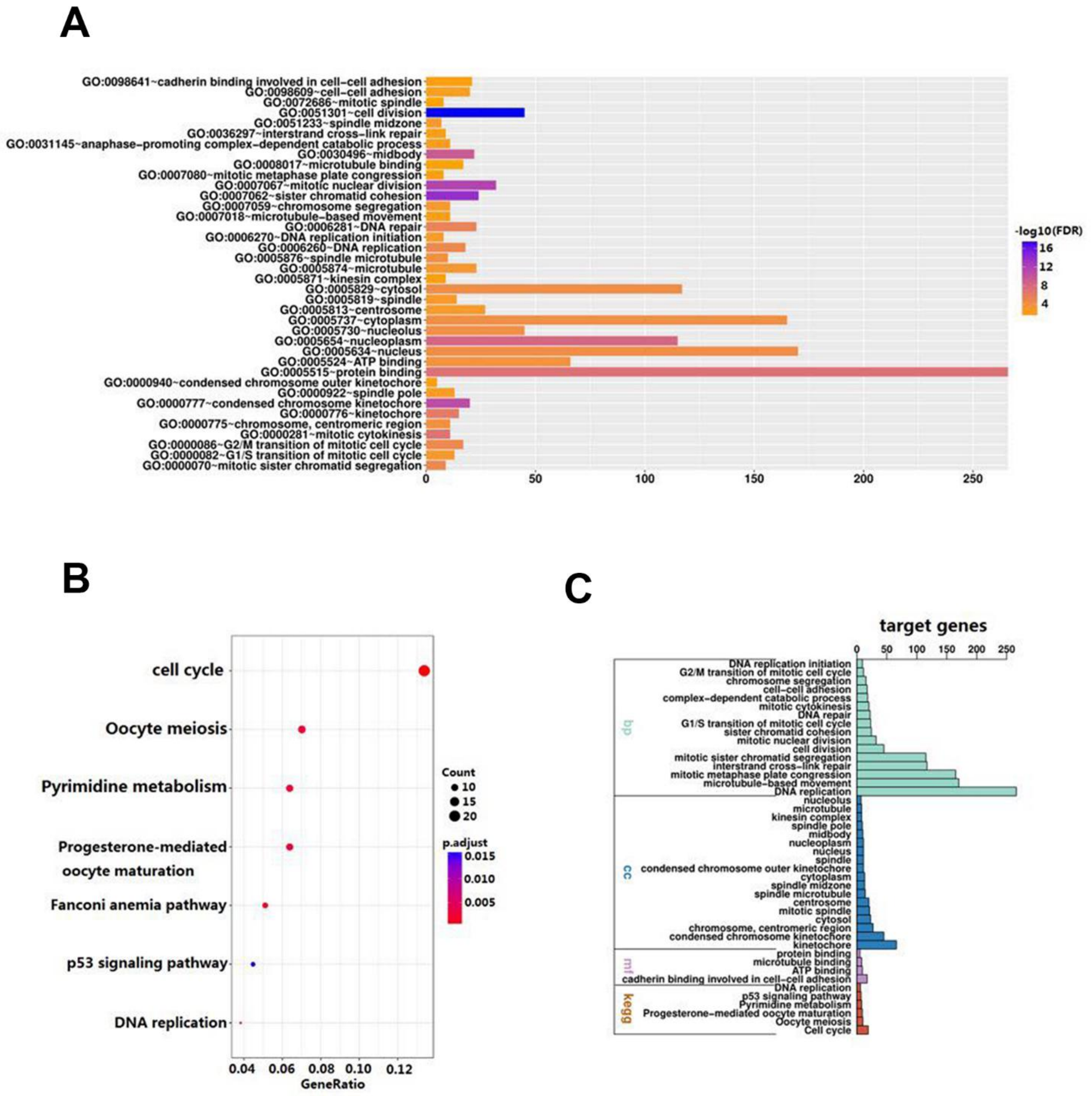

Supplementary Figure 4. Protein-coding genes co-expressed with AC006504.8 lncRNA enriched by significant KEGG pathways and GO-based functional enrichment analysis. (**A**) The 38 GO processes enriched among 495 DPCG targets of AC006504.8 lncRNA. (**B**) The 6 KEGG pathways for AC006504.8 lncRNA-related DPCGs. (**C**) Number of target genes enriched (the horizontal axis represented the respective -Log10 (FDR) of different paths and the vertical axis denoted the path name; bp: Biology Process, cc: Cellular Components; mf: Molecular Function).

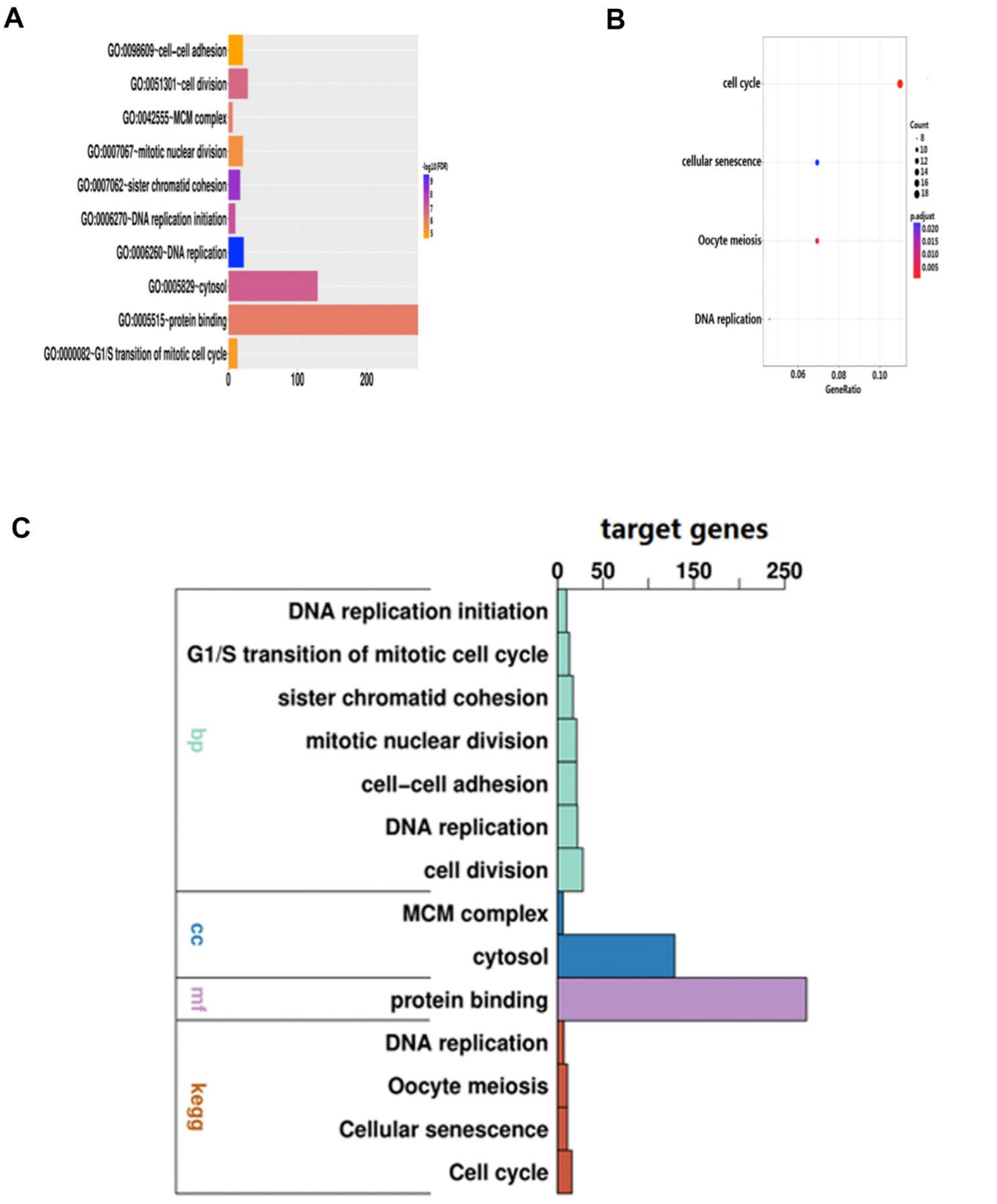

Supplementary Figure 5. Protein-coding genes co-expressed with AC090114.2 lncRNA enriched by significant KEGG pathways and GO-based functional enrichment analysis. (**A**) The 10 GO processes enriched among 552 DPCG targets of AC090114.2 lncRNA. (**B**) The 4 KEGG pathways for AC090114.2 lncRNA-related DPCGs. (**C**) Number of target genes enriched (the horizontal axis represented the respective -Log10 (FDR) of different paths and the vertical axis denoted the path name; bp: Biology Process, cc: Cellular Components, mf: Molecular Function).

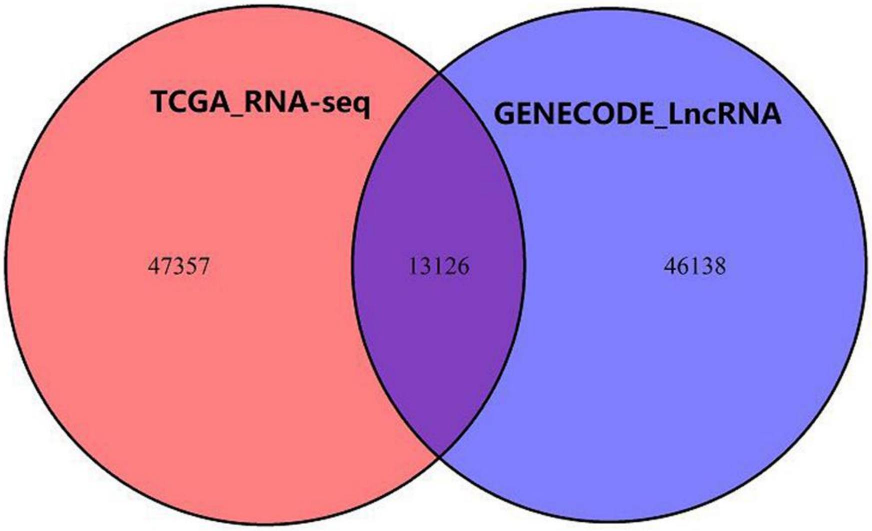

Supplementary Figure 6. After 60483 RNA-ENSG_ID and 59264 lncRNA-ENSG_ID were intersected to obtain 13126 lncRNA-Gene_name for this study.
