# Supplementary material for: A novel five-lncRNA signature panel improves high-risk survival prediction in patients with cholangiocarcinoma
Source: Aging (Albany NY). 2021 Jan 20;13(2):2959–81. doi: 10.18632/aging.202446 (PMC7880389; doi:10.18632/aging.202446)
Supplement: Supplementary Tables [file aging-13-202446-s002.pdf]

SUPPLEMENTARY TABLES

Supplementary Table 1. KEGG pathway analysis of predicted targets from the 5-lncRNA.

Pathway	Count	Genes	FDR
**hsa04110:Cell cycle**	10	BUB1; CCNB2; CDC20; CDC45; MAD2L1; MCM4; ORC6; PKMYT1;PLK1;PTTG1	P<0.001
**hsa04114:Oocyte meiosis**	7	BUB1; CCNB2; CDC20; PKMYT1; PLK1; PTTG1; MAD2L1	0.002990543
**hsa03460:Fanconi anemia pathway**	5	BLM; FAAP24; FANCG; FANCI; RAD51	0.002990543
**hsa03030:DNA replication**	4	FEN1; MCM4; POLA2; RFC4	0.005179535
**hsa04914:Progesterone-mediated oocyte maturation**	5	PKMYT1; PLK1; BUB1; CCNB2; MAD2L1	0.027335116

Supplementary Table 2. Results of high- and low-risk score groups gene set enrichment analysis (GSEA) analysis of the predicted core target genes.

Risk score groups	Pathway	Core enrichment (YES) genes	p-value	FDR
**Low risk**	KEGG_T_CELL_RECEPTOR_SIGNALING_PATHWAY	LAT; MAPK3; JUN; IL10; CTLA4; FOS; NFKBIE; CBL; RAF1; CARD11; CHP2; CD4; CD3G; RASGRP1; CSF2	<0.001	0.014
	KEGG_NEUROTROPHIN_SIGNALING_PATHWAY	MAPK10; NTF4; NFKBIE; RAF1; RIPK2; YWHAZ; MAP3K5; SH2B2; MAPK3; JUN; BCL2; NTRK3	<0.001	0.029
	BIOCARTA_KERATINOCYTE_PATHWAY	RAF1; MAP3K5; MAPK3; JUN; TNFRSF1B; BCL2; FOS	0.002	0.007
	BIOCARTA_IL2RB_PATHWAY	SYK; SOCS3; CBL; RAF1; JAK3; IL2RG; MAPK3; BCL2; FOS	0.008	0.014
**High risk**	BIOCARTA_COMP_PATHWAY	C3; C4B; MASP2; C9; C6; C5; C2; C4A; C8A; CFB; C1S; C1R; MBL2; MASP1	0.010	0.034

Supplementary Table 3. The clinicopathological characteristics of the TCGA cohort and WMU cohort.

Clinicopathological characteristics	Discovery cohort (n=36)	Validation cohort (n=90)	χ2	*p*-value
Age			0.59	0.441
<60	9	33		
≥60	22	57		
Gender			0.20	0.652
Male	20	46		
Female	16	44		
Stage			0.79	0.375
Stage I-II	23	59		
Stage III-IV	8	31		
Grade			0.31	0.578
Grade 1-2	10	40		
Grade 3-4	19	50		
Relative family cancer history			0.02	0.900
Yes	19	43		
No	10	24		
Primary pathology residual tumor			1.6	0.205
R1	4	18		
R0	25	53		
BMI			1.21	0.271
>25	23	57		
≤24.9	8	33		
Recurrence			0.60	0.440
Yes	15	52		
No	10	24		
History Hepatoma risk factors			1.82	0.177
Yes	14	45		
No	21	39		
Cancer status			1.57	0.21
With tumor	17	64		
Tumor free	12	26		
Primary pathology histological type			0.66	0.416
Intrahepatic	25	66		
Other	6	24		
Postoperative radiotherapy			0.47	0.495
Yes	8	24		
No	20	43		
Five lncRNA Risk score			1.05	0.306
High	18	54		
Low	18	36		
